# Environmental Enrichment Enhances Episodic-Like Memory in Association with a Modified Neuronal Activation Profile in Adult Mice

**DOI:** 10.1371/journal.pone.0048043

**Published:** 2012-10-22

**Authors:** Marianne Leger, Anne Quiedeville, Eleni Paizanis, Sharuja Natkunarajah, Thomas Freret, Michel Boulouard, Pascale Schumann-Bard

**Affiliations:** Université de Caen Basse-Normandie, Groupe Mémoire et Plasticité comportementale (GMPc), EA 4259, Caen, France; Tulane University Medical School, United States of America

## Abstract

Although environmental enrichment is well known to improve learning and memory in rodents, the underlying neuronal networks' plasticity remains poorly described. Modifications of the brain activation pattern by enriched condition (EC), especially in the frontal cortex and the baso-lateral amygdala, have been reported during an aversive memory task in rodents. The aims of our study were to examine 1) whether EC modulates episodic-like memory in an object recognition task and 2) whether EC modulates the task-induced neuronal networks. To this end, adult male mice were housed either in standard condition (SC) or in EC for three weeks before behavioral experiments (n = 12/group). Memory performances were examined in an object recognition task performed in a Y-maze with a 2-hour or 24-hour delay between presentation and test (inter-session intervals, ISI). To characterize the mechanisms underlying the promnesiant effect of EC, the brain activation profile was assessed after either the presentation or the test sessions using immunohistochemical techniques with c-Fos as a neuronal activation marker. EC did not modulate memory performances after a 2 h-ISI, but extended object recognition memory to a 24 h-ISI. In contrast, SC mice did not discriminate the novel object at this ISI. Compared to SC mice, no activation related to the presentation session was found in selected brain regions of EC mice (in particular, no effect was found in the hippocampus and the perirhinal cortex and a reduced activation was found in the baso-lateral amygdala). On the other hand, an activation of the hippocampus and the infralimbic cortex was observed after the test session for EC, but not SC mice. These results suggest that the persistence of object recognition memory in EC could be related to a reorganization of neuronal networks occurring as early as the memory encoding.

## Introduction

Environmental enrichment consists of complex housing conditions enhancing sensory-motor activities, social interactions and cognitive stimulations [1], and therefore stimulating brain plasticity. This enriched condition (EC) is well known to reduce aged-related cognitive deficits of rodents and to alleviate those occurring in models of neurodegenerative disorders (for review, see [2] and [3]). EC also improves cognitive abilities in healthy adult mouse (animals without particular deficits) although this aspect was less studied. Indeed, EC is associated with an improvement of spatial [4], [5] but also non-spatial learning and memory performances [6], [7], [8].

EC is thus an interesting model to elicit and understand the mechanisms underlying brain plasticity in laboratory animals. Brain neurobiological changes associated with beneficial effects of EC on memory have been extensively studied. Many reports show an increase of weight and thickness of the cortex and of the hippocampus volume (a brain region that is particularly involved in learning and memory) [9], [10], [11]. Hippocampus neurogenesis and synaptogenesis enhancement have also been proposed as neurobiological changes supporting beneficial effects of EC on memory [3], [5]. While these brain morphological and structural changes (particularly those occurring in the hippocampus) are well documented, their functional consequences on the brain circuitry underlying memory storage have been less explored.

Indeed, it is established that neuronal plasticity occurs during memory tasks, but whether they could be part of the mechanisms underlying the beneficial effects of EC remains an open question. We have previously shown that the beneficial effects of EC on recent memory in an aversive memory task were associated with a higher activation of the frontal cortex [6]. Here we focus on the effect of EC on episodic-like memory in mice. Several studies revealed beneficial effects of EC on this type of memory using an object recognition task. The advantage of this behavioral paradigm relies on the absence of positive or negative reinforcers and on the possibility of retention interval manipulation. Moreover, memory assessment is based on the natural exploration of a novel object performed in a familiar, non-stressing environment, and does not require extensive training, nor rule learning. In the literature, higher novel object discrimination performances in EC mice compared to littermates housed in standard condition (SC) were reported, even after long (24 h, 48 h) inter-session intervals (ISI). SC mice were not able to discriminate the novel object at these long ISI [7], [8], [12]. Some mechanisms on this type of memory have been proposed to underlie these EC-induced benefits, such as an increased hippocampal synaptic density [7], or an enhancement of hippocampal neurogenesis leading to long-term memory prolongation [13]. However, as pointed above, the potential modulation of neuronal activity during memory task has not been described in EC. The neuronal networks supporting memory functioning have been extensively studied, with a particular emphasis on the neurocircuitry of episodic-like memory in the object recognition task. Among the brain structures known to be involved in this memory, the hippocampus and the perirhinal cortex have been particularly studied [14], [15]. The integrity of the hippocampus [16] and the perirhinal cortex [17], [18], [19], [20] were required for the components of the episodic-like memory assessed through object recognition task. Higher neuronal activation in the perirhinal cortex in association with regional activation in the CA1 and CA3 areas of the hippocampus were observed after a novel object discrimination task in functional activation studies [21], [22], suggesting a functional role of both structures in object recognition.

Thus, we aimed at determining whether the beneficial effects of EC on episodic-like memory were associated with a modification of the brain activation profile at both the presentation and the test sessions. To this end, we first characterized the duration of object recognition memory trace in SC mice in a new object recognition paradigm performed in a Y-maze adapted from Dellu and colleagues [23]. Different ISI were used, ranging from 2 hours to 24 hours. Second, we assessed the effects of EC on memory performances tested with two ISI for which SC mice are able, or unable, of discriminating a novel object (respectively: 2 h and 24 h-ISI). These two ISI were chosen according to the results of the first experiment in order to dissociate differential effects of EC on memory expression and on memory trace prolongation respectively. Based on the memory trace prolongation elicited by EC (24 h-ISI), we explored the neuronal activation profile related to the memory task by immunohistochemistry using c-Fos expression as a neuronal activation marker of memory formation [24].

## Materials and Methods

### Animals

NMRI male mice, 10 weeks of age at the beginning of the experiments, were used (local breeding facility, F1 from Centre d'Elevage René Janvier, Le Genest, France). All animals were maintained in a room with reversed 12 h light-dark cycle (20∶00–8∶00), at constant temperature (21°C) and humidity (55%). Water and food were available *ad libitum*. All experiments were carried out in accordance with the European Communities Council Directive (2010/63/UE) regarding the care and use of animals for experimental procedures, and approved by the regional ethical committee (Comité d'Ethique NOrmandie en Matière d'EXpérimentation Animale, CENOMEXA) (agreement number: 01-10-09/17/07-12).

### Environmental enrichment

For each experiment, mice were randomly assigned to either SC or EC housing. SC mice were maintained in transparent polycarbonate cages (42×29×15 cm^3^, 6 mice per cage) containing nesting material and a cardboard tube. Mice from the EC groups were continuously housed in environmental enrichment using large polycarbonate cages (80×60×60 cm^3^, 12 mice per cage), provided with various objects of different shapes, sizes, colors, textures and material (wood, plastic and metal) and a large running wheel, as already described [6]. To ensure novelty, most of the objects and their location were changed twice a week. To limit inter-individual male antagonistic behaviors [25], some objects were neither changed nor cleaned, and used nesting material was placed in the cage at each cage cleaning. Mice were placed continuously in SC (n = 12) or EC (n = 12) for three weeks before memory testing and maintained in these conditions during behavioral experiments. A three-week duration of EC is indeed already known to improve memory [6].

### Behavioral experiments

Behavioral experiments were conducted between 8∶30 and 12∶30. Mice were placed in the experimental room 30 minutes before the beginning of the behavioral experiments.

#### Object recognition in a Y-maze

The apparatus consisted of a gray plastic Y-maze with three arms (33×8×16 cm^3^ each). The object recognition paradigm was adapted from Arque and collaborators [26] and performed in a Y-maze as described in the literature for rats [23]. During the presentation session, two identical objects were placed in the maze, one in the distal area of two arms. The mouse was placed in the starting arm containing no object, faced to the wall and was allowed to freely explore the apparatus and the objects. Object exploration was considered when the nose of the animal was directed towards the object at a distance below 2 cm. When a criterion of 20 seconds of total exploration time of both objects was achieved, the mouse was returned to its respective SC or EC home cage until the test session. This criterion was selected according to the protocol used by Arque and collaborators [26]. During the test session, a third copy of the two familiar objects and a novel object were placed in the maze. Again, the mouse was allowed to freely explore the three arms and the objects until the criterion of 20 seconds of exploration of both objects was reached. The nature of the objects (Lego® *vs* Falcon®) and the position of the novel object (left *vs* right) were randomized. The time spent to reach the criterion was measured and the total number of entries in the three arms was recorded and used as an index of locomotor activity. The time of exploration of each object was manually recorded and used as an index of memory performances by comparison with the chance level (10 seconds). Percentages of entries and time spent in the two arms containing the objects were compared to the chance level (50%). Mice failing to reach the criterion within 10 minutes were excluded from the analysis.

#### Characterization of memory trace duration in SC mice

A first experiment was conducted to assess the effect of increasing ISI on object recognition memory. Adult mice were maintained in SC and tested in the object recognition task using increasing ISI: 2 h-ISI (n = 12), 4 h-ISI (n = 12), 6 h-ISI (n = 12) and 24 h-ISI (n = 12).

#### Effects of EC on object recognition memory performances

We assessed the ability of EC to either improve existing memory performances of SC mice (2 h-ISI; n = 12 per housing condition) or to extend memory trace persistence (24 h-ISI; n = 12 per housing condition). These two ISI were chosen according to the results of the first experiment.

### Brain activation experiments

**Figure 1:**
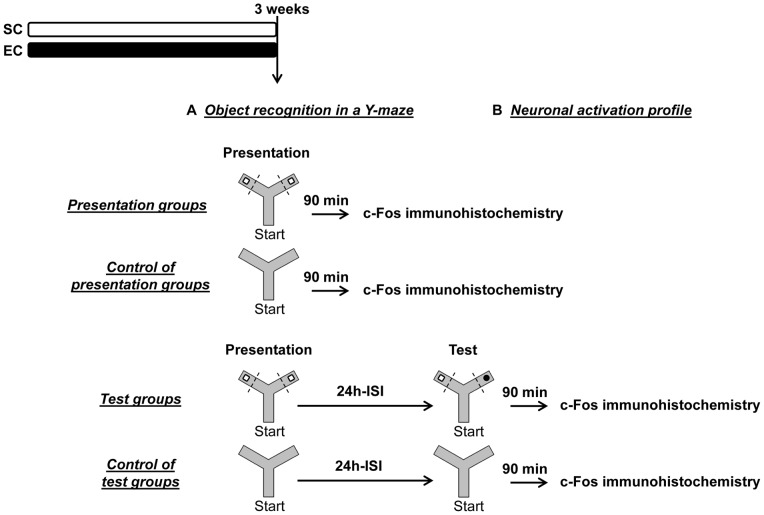
Brain activation experimental design. A. After 3 weeks in either standard condition (SC) or enriched condition (EC), memory performances of mice were tested in an object-recognition task performed in a Y-maze with an inter-session interval (ISI) of 24 h (“Test” groups, n = 12 for each housing condition). **B.** Ninety minutes after the presentation or after the test session, animals were euthanized and the brains were collected for c-Fos immunohistochemistry (“Presentation” groups and “Test” groups, n = 10 per housing condition). Additional mice were exposed to the same behavioral procedures but without any object (“Control of presentation” groups and “Control of test” groups, n = 8 and n = 10 per housing condition respectively).

To assess the neuronal activation profile related to the memory task (24 h-ISI), mice (n = 10 SC and n = 10 EC, for the presentation group and for the test group) were euthanized by cervical dislocation after 90 minutes, a time that allows the measurement of peak c-Fos immunoreactivity [27]. The brains were rapidly removed, immersed a few seconds in isopentane (−40°C) and stored at −80°C until c-Fos immunohistochemistry was processed. In order to control for non-memory aspects of the behavioral task, such as locomotor activity or context arousal, additional SC and EC mice (n = 8 “control of presentation” mice and n = 10 “control of test” mice per housing condition) were exposed to the same behavioral procedure, but in absence of the critical learning stimulus, *i.e*. the objects, during both sessions (Figure 1). During the presentation session, control mice were exposed 200 seconds to the apparatus, while they were exposed to 300 seconds to the apparatus during the test session. Determination of this time of exposure to the Y-maze was based on the mean time to reach the criterion in SC and EC mice during each session in a preliminary study (data not shown). Using exposed control mice is a standard protocol for controlling brain activation related to several memory tasks [28], [29], [30], including object recognition memory tasks [31].

Brain coronal sections (20 µm thickness) were obtained by cryosectioning. The distance between sections was 100 µm along the whole brain. It was reduced to 60 µm for the smaller brain structures such as the frontal and prefrontal cortices. Sections were mounted on gelatinized slides and stored at −80°C until processing c-Fos immunohistochemistry.

According to the method described by Sundquist and Nisenbaum [32], slides were first fixed in a solution of 4% paraformaldehyde for 10 minutes at room temperature before being rinsed several times in 0.1% Triton X-100/PBS 0.1 M solution. Slides were then immersed for 15 minutes in 3% H_2_O_2_/10% methanol/PBS 0.1 M to quench endogenous peroxidase. After several washes, sections were incubated in a 10% solution of donkey serum/1% Bovine Serum Albumin (BSA)/0.1% Triton X-100/PBS 0.1 M before incubation during 90 minutes in a solution of 1% BSA/0.1% Triton X-100/PBS 0.1 M supplemented by the primary antibody (rabbit anti-c-Fos IgG, 1∶300, SC 7202, Santa-Cruz Biotechnology, Santa-Cruz, CA). After several rinses, sections were incubated for 30 minutes at room temperature with the secondary antibody (biotinylated goat anti-rabbit IgG, 1∶200, Vector Laboratories, France) and finally stained using ABC staining system (Vectastain ABC, Elite kit, Vector Laboratories, France). Peroxidase activity was detected by incubation of sections for 5 minutes in 0.05% Diaminobenzidine/0.2% nickel-ammonium-sulphate/0.01% H_2_O_2_/PBS 0.1 M. To avoid staining variability, sections of test or presentation groups and respective control groups of each housing condition were treated in the same incubation bath. Sections were rinsed, dehydrated and coverslipped with R.A. Mounting Medium™ (Richard-Allan Scientific, Kalamazoo, MI, USA).

Sections were analyzed by an experimenter blind to the group. Quantification of c-Fos-positive nuclei was manually performed on an Olympus microscope (x20 – x40 objectives) on eight consecutive sections bilaterally analyzed for each brain region considered. According to the Paxinos and Franklin's atlas [33], several brain regions were selected: the frontal cortex, the median prefrontal cortex (prelimbic, infralimbic and anterior cingulate cortices), the hippocampus, the perirhinal cortex and the baso-lateral amygdala. The total number of c-Fos-positive nuclei was then determined in each region of interest. This number was thereafter expressed as a percentage of the mean number of c-Fos-positive nuclei of corresponding control animals (SC or EC, presentation or test session) for the brain region considered [29].

### Statistical Analysis

Values are expressed as mean ± SEM. Statistical analyses were processed with Statview 5.0® software. Analyses of variance (ANOVA) with the housing condition (SC or EC) as the independent variable and with the time spent to reach the criterion or the total number of entries during the presentation and the test session and the brain structure selected for analysis of c-Fos-positive cells, as the repeated measure, were performed. When appropriate, *post hoc* Student-Newman-Keuls (SNK) multiple range tests were used. Because the times spent to explore the objects during the test session were dependent of each other, a univariate *t*-test was used to compare the exploration time of the novel object to the chance level of exploration (10 seconds), as seen in the literature [34]. The percentages of entries and of time spent in the arm containing the novel object were compared with 50%. The number of c-Fos-positive cells was also compared to respective control groups (100%) with a univariate *t*-test. Finally, the correlation between the activity of the hippocampus and the activity of the other brain regions was analyzed using a test of Pearson's correlation coefficient. A significant difference was considered when the *P* value was lower than 0.05.

## Results

### SC mice performed the object recognition memory task in a time-dependent manner

**Figure 2:**
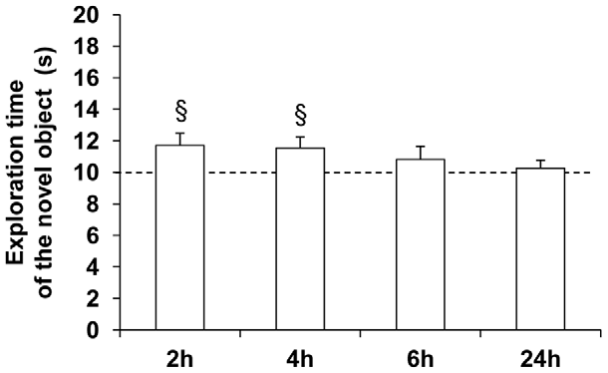
Effect of increasing ISI on object recognition memory performances in mice housed in SC. Data are expressed as the mean exploration time (± SEM) of novel object for the test session with increasing ISI: 2 h (n = 10), 4 h (n = 11), 6 h (n = 11) and 24 h (n = 12). Exploration time of the novel object was significantly higher than the chance level (10 seconds) with 2 h and 4 h-ISI, suggesting that SC mice discriminated the novel object at both delays (univariate *t*-test: § denotes *P*<0.05).

Results indicate that all mice spent similar time exploring the two objects during the presentation session (data not shown, univariate *t*-test: 2 h-ISI, t(9) = −0.21; 4 h-ISI, t(10) = −0.94; 6 h-ISI, t(10) = 0.00 and 24 h-ISI, t(11) = −2.00, *P*>0.05 for each ISI compared to the chance level (10 seconds)). By contrast, during the test session occurring at 2 h and 4 h-ISI, mice significantly spent more time exploring the novel object compared to the chance level (univariate *t*-test: 2 h-ISI, t(9) = 2.33, *P*<0.05 and 4 h-ISI, t(10) = 2.37, *P*<0.05 compared to the chance level (10 seconds)) (Figure 2). No discrimination of the novel object was observed in groups submitted to longer ISI (univariate *t*-test: t(10) = 1.03 and t(11) = 0.51, *P*>0.05 compared to the chance level (10 seconds) for 6 h-ISI and 24 h-ISI respectively). The time spent exploring the novel object did not differ between groups (ANOVA: *F*_(3;40)_ = 1.03, *P*>0.05).

### EC did not modify object recognition memory performances with 2 h-ISI

During the presentation session, all groups exhibited similar exploration time for the two identical objects (data not shown, univariate *t*-test: SC, t(10) = 2.21, *P*>0.05 and EC, t(8) = 0.89, *P*>0.05 compared to the chance level (10 seconds)).

**Figure 3:**
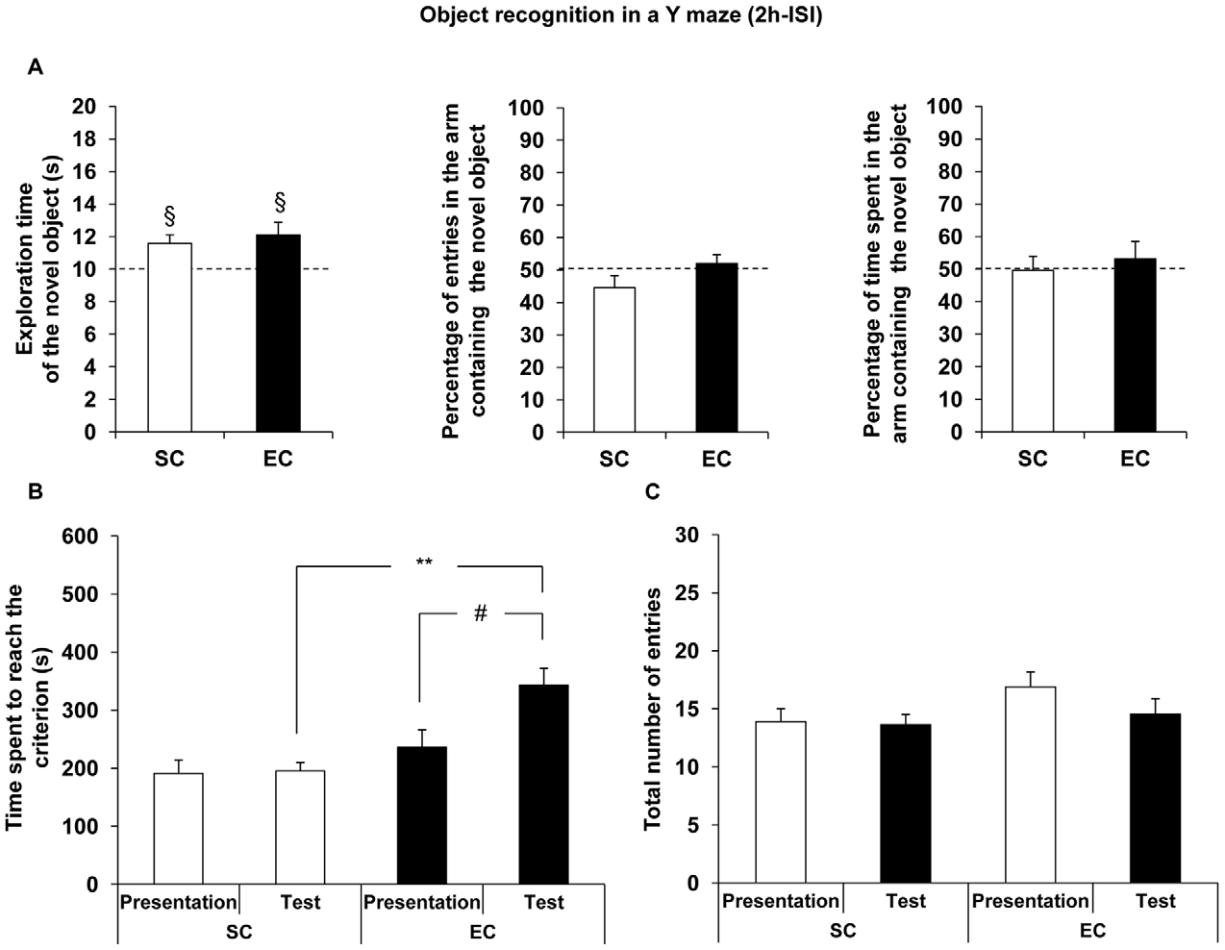
Effect of EC on object recognition memory tested with 2 h-ISI. A. Data are the mean exploration time (± SEM) of the novel object during the test session in SC (n = 11) and EC (n = 9) groups. Exploration time of the novel object was significantly higher than the chance level (10 seconds) in the two groups (univariate *t*-test: § denotes *P*<0.05 for SC mice and EC mice), suggesting that all mice discriminated the novel object. Neither the percentage of entries, nor the percentage of time spent in the arm containing the novel object were different from the chance level (50%) in both groups. **B.** The time spent to reach the criterion (20 seconds of total exploration of the objects) did not differ between groups during the presentation session. By contrast, during the test session, the time spent to reach the criterion was significantly higher in EC mice than in SC ones (ANOVA with repeated measurements followed by a SNK multiple range test: ** denotes *P*<0.01, significantly different from SC group). The time spent to reach the criterion was significantly different between two sessions in EC group (ANOVA: *#* denotes *P*<0.05, significantly different from the presentation session). **C.** The total number of entries in the three arms did not differ between groups.

By contrast, during the 2 h-ISI test session, the exploration time of the novel object was significantly different compared to the chance level (10 seconds), whatever the group considered (univariate *t*-test: t(10) = 2.85, *P*<0.05 and t(8) = 2.95, *P*<0.05 in SC and EC mice, respectively) (Figure 3A). Besides, no modification of the percentages of entries and of time spent in the arm containing the novel object were observed (respectively: SC, t(10) = −1.54; EC, t(8) = 0.81, *P*>0.05 and SC, t(10) = −0.09; EC, t(8) = 0.61, *P*>0.05; univariate *t*-test compared to the chance level (50%)).

The time spent to reach the criterion did not differ between groups during the presentation session, while, during the test session, EC mice spent significantly more time to reach a total of 20 seconds of object exploration compared to SC mice (ANOVA with repeated measurements: housing condition effect, *F*_(1;18)_ = 16.23, *P*<0.001; session effect, *F*_(1;18)_ = 5.83, *P*<0.05 and interaction, *F*_(1;18)_ = 5.16, *P*<0.05 followed by a SNK multiple range test: housing condition effect, *P*<0.01 for the test session). In fact, SC mice spent similar time to reach the criterion during both sessions (ANOVA: *F*_(1;10)_ = 0.04, *P*>0.05). By contrast, EC mice spent significantly more time to reach the criterion during the test session compared to the presentation one (ANOVA: *F*_(1;8)_ = 5.34, *P*<0.05), suggesting a potential habituation to the presence of the objects between the two sessions in these animals (Figure 3B). Finally, the total number of entries in the three arms did not differ between groups, whatever the session considered (ANOVA with repeated measurements: no housing condition effect *F*_(1;18)_ = 2.90, no session effect *F*_(1;18)_ = 1.67, and no interaction *F*_(1;18)_ = 1.05), suggesting no modulation of locomotor activity by EC (Figure 3C).

### EC prolonged object recognition memory trace with 24 h-ISI

No significant difference of the exploration time of each object was found during the presentation session, whatever the housing condition (data not shown, univariate *t*-test: SC, t(9) = 0.56, *P*>0.05 and EC, t(9) = −0.61, *P*>0.05 compared to the chance level (10 seconds)).

**Figure 4:**
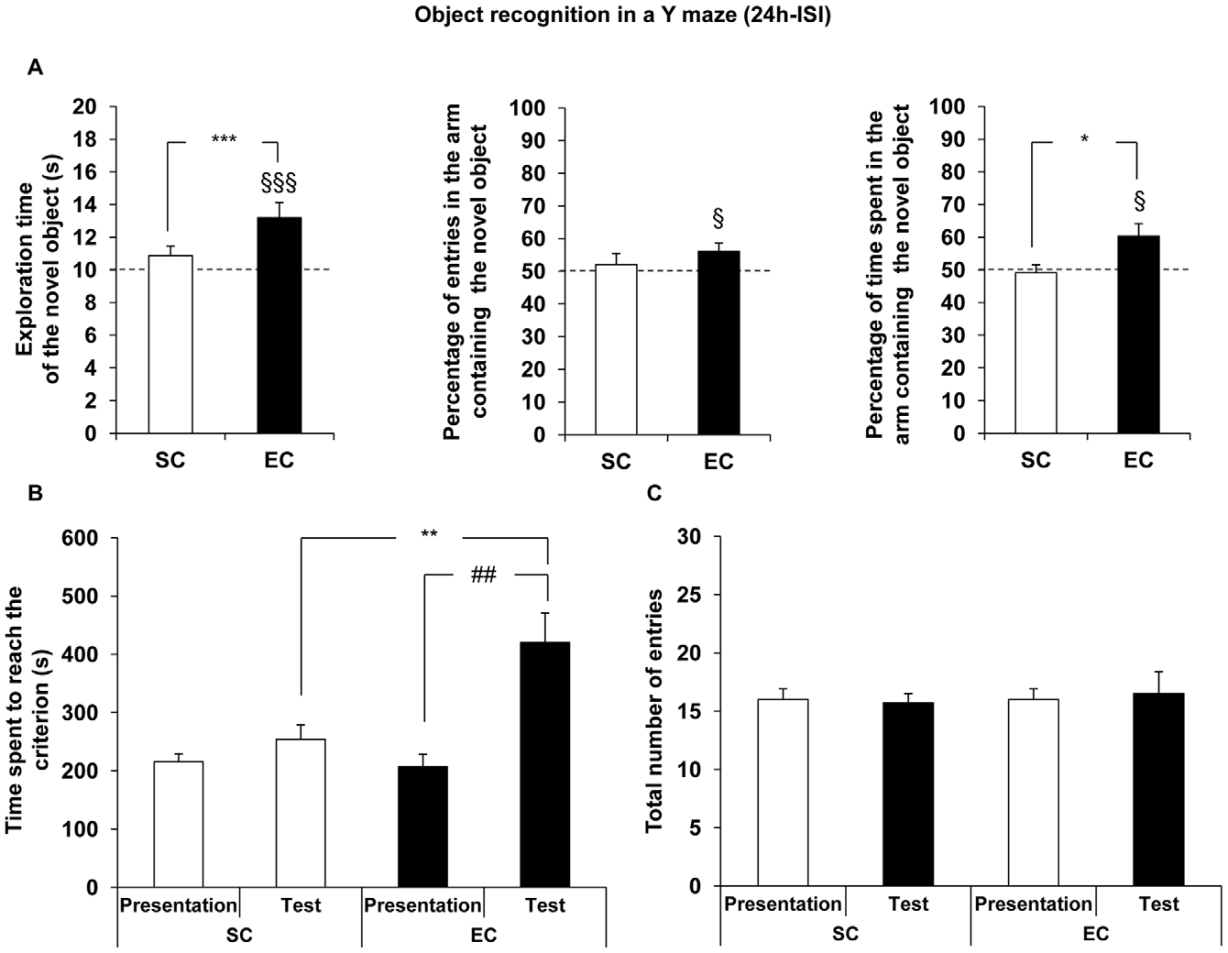
Effect of EC on object recognition memory tested with 24 h-ISI. A. Data are the mean exploration time (± SEM) of the novel object during the test session in SC (n = 10) and EC (n = 10) groups. Only EC mice discriminated the novel object compared to the chance level (10 seconds) (univariate *t*-test: §§§ denotes *P*<0.001). Moreover, the time spent to explore the novel object was significantly higher in EC mice compared to SC ones (ANOVA: *** denotes *P*<0.001). The percentage of entries and the percentage of time spent in the arm containing the novel object were significantly different from the chance level (50%) in EC group only (univariate *t*-test: § denotes *P*<0.05). A significant difference of the percentage of time spent in the arm containing the novel object was observed between groups (ANOVA: * denotes *P*<0.05). **B.** The time spent to reach the criterion did not differ between groups during the presentation session but was significantly higher in EC mice during the test session (ANOVA with repeated measurements followed by a SNK multiple range test: ** denotes *P*<0.01, significantly different from SC group). EC mice spent significantly more time reaching the criterion during the test session (ANOVA: *##* denotes *P*<0.01, significantly different from the presentation session). **C.** The total number of entries in the three arms did not differ between groups.

At the 24 h-ISI test session, EC mice displayed a significantly longer time of novel object exploration compared to the chance level (10 seconds; univariate *t*-test: t(9) = 6.19, *P*<0.001), in contrast to the SC mice (univariate *t*-test: t(9) = 1.17, *P*>0.05). Time exploring the novel object significantly differed between groups (ANOVA: *F*_(1;18)_ = 18.44, *P*<0.001) (Figure 4A). Similarly, a significant difference from chance level (50%) was observed in EC, but not SC mice, for the percentages of entries and of time spent in the arm containing the novel object (respectively: SC, t(9) = 0.68, *P*>0.05; EC, t(9) = 2.59, *P*<0.05 and SC, t(9) = −0.44, *P*>0.05; EC, t(9) = 2.85, *P*<0.05). The percentage of time spent in the arm containing the novel object was significantly higher in the EC group (ANOVA: *F*_(1;18)_ = 7.01, *P*<0.05) than in the SC one.

As in the previous experiment, the time spent to reach the criterion was significantly higher in EC mice compared to SC ones during the test session, but not during the presentation (ANOVA with repeated measurements: housing condition effect, *F*_(1;18)_ = 12.79, *P*<0.01; session effect, *F*_(1;18)_ = 13.26, *P*<0.01 and interaction, *F*_(1;18)_ = 16.30, *P*<0.001 followed by a SNK multiple range test: no housing condition effect, *P*>0.05, and a housing condition effect, *P*<0.01, for the presentation and the test sessions, respectively). A significant difference in the time spent to reach the criterion between the two sessions was only found in EC mice (ANOVA: SC, *F*_(1;9)_ = 0.28, *P*>0.05 and EC, *F*_(1;9)_ = 17.16, *P*<0.01), suggesting again an habituation to the objects between the two sessions in EC animals (Figure 4B). The total number of entries in the three arms did not differ between groups, whatever the session considered (ANOVA with repeated measurements: no housing condition effect, *F*_(1;18)_ = 0.15, no session effect, *F*_(1;18)_ = 0.00, and no interaction *F*_(1;18)_ = 0.05), suggesting no modulation of locomotor activity by EC (Figure 4C).

Finally, we verified that the “presentation” groups of each housing condition (*i.e*., animals submitted to the presentation of the apparatus with objects) explored equally the two identical objects (data not shown, univariate *t*-test: SC, t(9) = 0.67, *P*>0.05 and EC, t(9) = −0.43, *P*>0.05, compared to the chance level (10 seconds)). In addition, in the control groups, we ensured that EC did not modify the locomotor activity in the Y-maze during both sessions. Indeed, the total number of entries in SC and EC mice of control groups did not differ, whatever the session considered (data not shown, ANOVA with repeated measurements: no housing condition effect, *F*_(1;18)_ = 3.24, no session effect, *F*_(1;18)_ = 1.39 and no interaction, *F*_(1;18)_ = 1.81).

Taken together, these results suggest that EC prolonged object recognition memory up to 24 h-ISI. In order to better understand the neuronal processes underlying these beneficial effects of EC on memory, we examined the potential modulation of the neuronal activation profile by EC related to either the presentation or the test session.

### Environmental enrichment modulated the neuronal activation profile related to the presentation session

The numbers of c-Fos-positive cells related to the presentation session in “control of presentation” groups in several brain regions (the frontal cortex, the prefrontal cortex, the hippocampus, the perirhinal cortex and the baso-lateral amygdala), did not differ between SC and EC mice (data not shown, ANOVA with repeated measurements, no housing condition effect *F*_(1,72)_ = 1.37, structure effect *F*_(6,72)_ = 22.66, *P*<0.001, and no interaction *F*_(6,72)_ = 2.18), suggesting no modulation of the neuronal activation profile during the apparatus exposure by EC.

**Figure 5:**
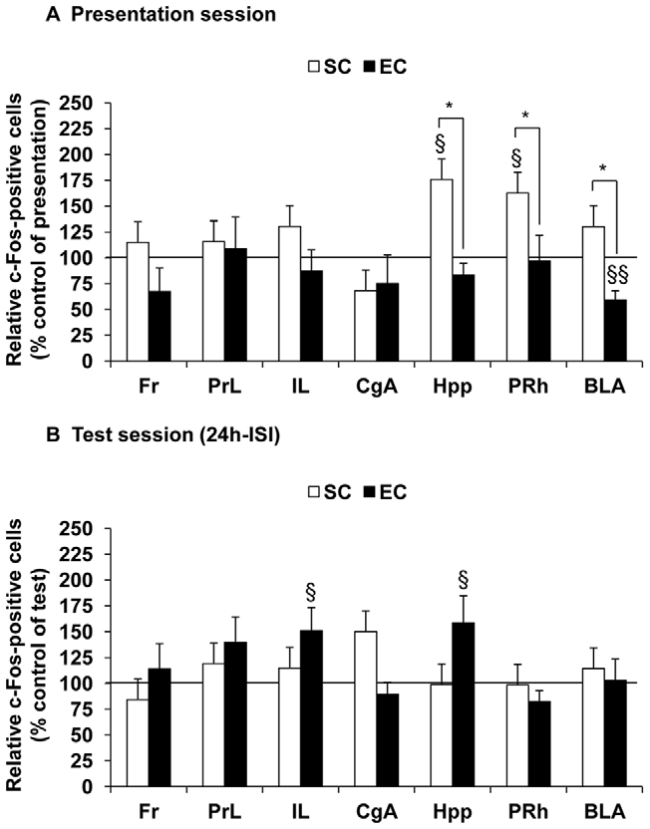
Effect of EC on neuronal activity in selected brain regions. Results are expressed as mean percentage changes of relative number of c-Fos-positive cells compared to control animals (which performed the task without any objects: n = 8 “control of presentation” animals per housing condition for the presentation session and n = 10 “control of test” animals per housing condition for the test session). **A.** A significant activation of the hippocampus and the perirhinal cortex was observed in SC mice during the presentation session while a significant reduction of activation of the baso-lateral amygdala occurred in EC mice (ANOVA with repeated measurements followed by a SNK multiple range test: * denotes *P*<0.05, significantly different from SC group; univariate *t*-test: § denotes *P*<0.05 and §§ denotes *P*<0.01, significantly different from respective “control of presentation” animals (100%)). **B.** The novel object discrimination during the test session (24 h-ISI) was associated with a significant activation of the infralimbic cortex and the hippocampus in EC mice (univariate *t*-test: § denotes *P*<0.05, significantly different from respective “control of test” animals (100%)). Abbreviations: Fr, frontal cortex; PrL, prelimbic cortex; IL, infralimbic cortex; CgA, anterior cingulate cortex; Hpp, hippocampus; PRh, perirhinal cortex; BLA, amygdala, baso-lateral nucleus.

**Figure 6:**
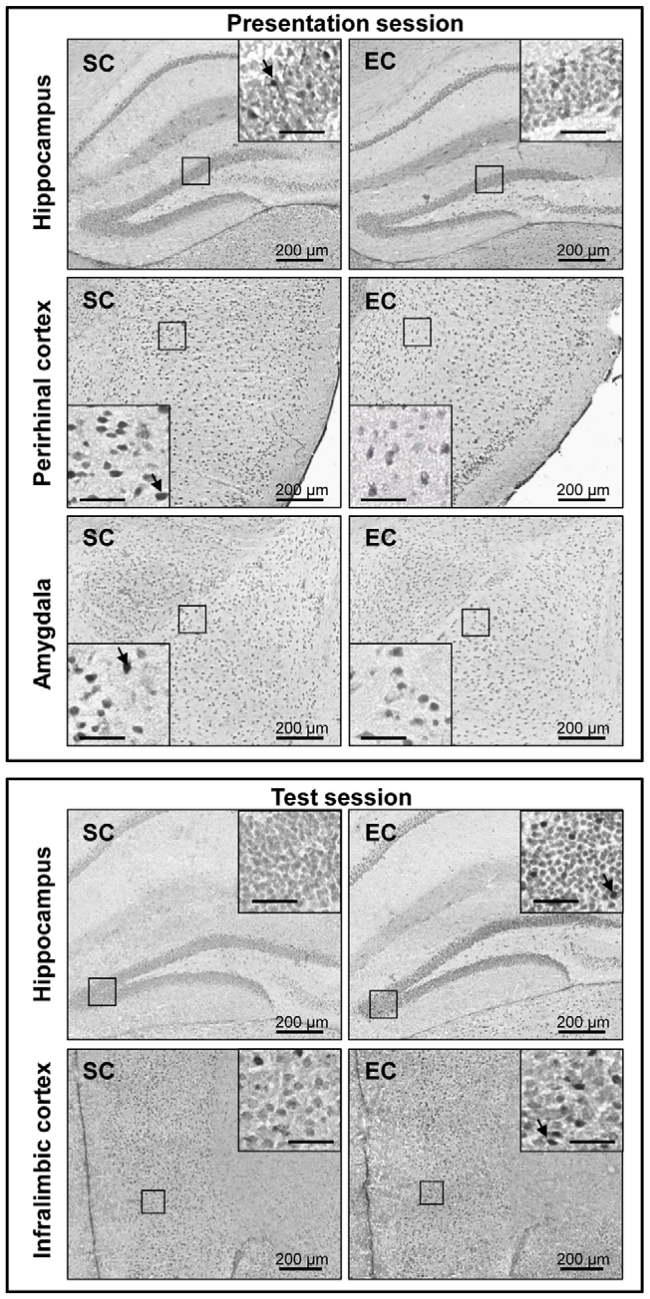
Microphotographs of brain c-Fos immunostaining related to the presentation and the test sessions. During the presentation session, the number of c-Fos-positive cells counted in the hippocampus (represented here by the granular cell layer of the dentate gyrus), the perirhinal cortex and the baso-lateral amygdala was lower in EC mice compared to SC ones. By contrast, the number of c-Fos-positive cells counted in the hippocampus and the infralimbic cortex during the test session was higher in EC mice. The dark arrows indicate examples of nuclei showing c-Fos immunoreactivity. Unannotated scale bars: 50 µm.

The neuronal activation profile related to the objects exploration during the presentation session is illustrated in Figure 5A (results are expressed as a percentage of the mean number of c-Fos-positive cells compared to “control of presentation” animals, which were not exposed to objects during the presentation session). Our results show a significant difference of neuronal activation between groups in three brain regions, namely the hippocampus, the perirhinal cortex and the amygdala (ANOVA with repeated measurements, no housing condition effect *F*_(1,96)_ = 3.91, structure effect *F*_(6,96)_ = 2.99, *P*<0.05, and interaction *F*_(6,96)_ = 2.31, *P*<0.05, followed by a SNK multiple range test: *P*<0.05, for the hippocampus, the perirhinal cortex and the baso-lateral amygdala). In fact, a significant neuronal activation occurred in the hippocampus and in the perirhinal cortex of SC mice (univariate *t*-test: t(9) = 2.35, *P*<0.05, for both structures compared to the respective “control of presentation” group (100%)), while a significantly reduced neuronal activation occurred in the baso-lateral amygdala of EC mice (univariate *t*-test: t(8) = −4.89, *P*<0.01 compared to the respective “control of presentation” group (100%)) (Figure 6).

### Novel object discrimination after 24 h-ISI by EC mice was associated with the activation of the hippocampus and infralimbic cortex

There was a significantly lower number of c-Fos-positive cells in the EC “control of test” group than in the respective SC “control of test” group (data not shown, ANOVA with repeated measurements, housing condition effect *F*_(1,96)_ = 6.83, *P*<0.05, structure effect *F*_(6,96)_ = 38.45, *P*<0.001, and no interaction *F*_(6,96)_ = 1.79).

When mice were exposed to the test session occurring after 24 h-ISI (*i.e*. in the presence of the novel object), no significant difference of the neuronal activation was observed between groups, whatever the brain structure considered (ANOVA with repeated measurements, no housing condition effect *F*_(1,90)_ = 0.88, structure effect *F*_(6,90)_ = 2.24, *P*<0.05, and interaction *F*_(6,90)_ = 3.12, *P*<0.01 followed by a SNK multiple range test, *P*>0.05, for each structure) (Figure 5B and Figure 6). However, a significant neuronal activation occurred in the hippocampus and the infralimbic cortex of EC mice (univariate *t*-test: t(9) = 2.35, *P*<0.05, for the hippocampus and t(9) = 2.41, *P*<0.05, for the infralimbic cortex, compared to the respective “control of test” group (100%)), which was not the case for SC mice that did not perform the task successfully. No modification of neuronal activity was detected in other brain regions.

### Hippocampus activity was correlated with the activity of several other regions in EC mice

Considering the involvement of the hippocampus in the object recognition memory and because the hippocampus neuronal activity was the most modulated by EC during both sessions of the memory task (*i.e*. an absence of hippocampal activation during the presentation session and a higher activation during the test session in EC mice compared to SC ones), we chose to examine the relation of the activity of this structure with the activity of the six other investigated brain regions. Interestingly, the correlation analysis indicated a significant correlation of the hippocampal activity with half of the regions of interest (especially the prefrontal cortex region) during the presentation session in EC mice, while only one correlation was found in SC mice (Table 1). Again, during the test session, the activity of half of the brain regions analyzed was significantly correlated with the hippocampus in EC mice, whereas no correlation was observed in SC mice.

**Table 1:** Interregional correlation matrix of the relative neuronal activity found in the hippocampus.

	SC group		EC group	
	Presentation	Test	Presentation	Test
Fr	0.10 (0.78)	0.46 (0.19)	0.39 (0.31)	0.09 (0.82)
PrL	0.56 (0.10)	0.31 (0.40)	0.67 (0.03*)	0.74 (0.02*)
IL	0.29 (0.43)	0.23 (0.54)	0.88 (0.0003***)	0.53 (0.15)
CgA	0.39 (0.27)	−0.31 (0.41)	0.81 (0.003***)	0.22 (0.62)
PRh	0.32 (0.38)	0.51 (0.14)	0.42 (0.23)	0.68 (0.03*)
BLA	0.79 (0.0043**)	0.13 (0.73)	0.46 (0.23)	0.75 (0.02*)

The first value indicates the calculated Pearson's correlation *r* value and the second value the exact *P* value corresponding to the correlation coefficient. Hippocampal activity was significantly correlated with more brain structures in EC mice than in SC ones during both the presentation and the test sessions (Same abbreviations as in Fig. 5; * denotes *P*<0.05, ** denotes *P*<0.01 and *** denotes *P*<0.001).

## Discussion

### Time-course of object recognition memory performed in a Y-maze in SC mice

Our behavioral results bring first evidence that mice housed in standard condition are able to discriminate a novel object when the memory task is performed in a Y-maze. Indeed, such a paradigm was reported in rats [23], [35], [36], [37], but, as far as we are aware, not in mice. This experimental protocol presents some advantages. Firstly, it requires only two brief sessions without a familiarization period, which facilitates the analysis of the brain activation profile related to either the presentation or to the test session. Moreover, these two sessions do not seem to lead to habituation to the apparatus, as no decreased exploration time was observed between the two sessions. Secondly, performing the memory task in a Y-maze allows the recording of other informative parameters, such as the time spent in the arm containing the novel object and the number of entries in this arm [23], which reflect exploration behavior. Thirdly, this object recognition memory task seems to be potentially more effortful. Indeed, as reported in a more complex paradigm where the two objects were also not visible from the starting arm [38], object discrimination is probably more based on recollection of past experiences than familiarity alone, which may be the case in the open-field apparatus. Finally, this task allowed us to detect beneficial effects of EC on memory in our conditions. This task was reported to be successfully performed in rats after a 2 h-ISI [23]. Here, we report the ability of our SC mice to discriminate the novel object for short ISI (2 h and 4 h), but not after longer ISI (6 h and 24 h). These results are in agreement with previous data of our laboratory indicating that mice were not able to discriminate a novel object after a 6 h-ISI in a paradigm led in open-field [39]. Our results show here evidence that the familiarization period (classically conducted on 3 consecutive days) is not essential to assess object recognition memory performances in our conditions, as previously reported by other authors [26]. We chose not to perform the familiarization period to the apparatus, based on preliminary results obtained in the open-field paradigm indicating a considerably reduced exploration activity in EC mice, leading to very poor exploration of objects compared to SC ones (data not shown). The reduction of the time spent exploring the objects by EC could be related to a faster habituation to novelty, as previously reported by others [40], [41], [42], [43], [44].

### Environmental enrichment enhanced object recognition memory persistence

In order to assess the effect of environmental enrichment on object recognition memory, we selected two delays of retention: the 2 h-ISI for which SC mice are able to discriminate the novel object and the 24 h-ISI, a delay for which SC mice did not successfully discriminate the novel object.

The performance at the 2 h-ISI indicates that both groups successfully and similarly performed the task. It was, however, not associated with higher percentages of entries, nor to time spent in the arm containing the novel object. In opposition to the literature [23], these additional parameters did not seem to be predictive of novel object discrimination in our conditions. Interestingly, EC mice needed more time to reach the criterion (*i.e*. time elapsed to obtain a total exploration of both objects for 20 seconds) during the test session. As the total number of entries is not different between groups for both sessions, it suggests that the environmental enrichment did not modify the locomotor activity in mice. Rather, the higher time spent to reach the criterion in EC mice could be related to a lowered interest for the objects, suggesting a faster habituation to novelty in these animals. The absence of memory improvement by EC after shorter ISI is not surprising and was reported in the literature [8], [12], [13]. Notably, it could be related to a ceiling effect with difficulty to reveal enhancement of memory performances. By contrast, when investigated with 24-ISI, object recognition memory was present in EC mice compared to SC ones. This is further supported by higher percentages of time and entries in the arm containing the novel object. Again, the time to reach the criterion was higher during the test session in EC mice, while there was no locomotor activity modulation. Interestingly, such a persistence of object recognition memory was also reported in the literature after longer ISI of 24 h and 48 h [8], [12], [13]. Some neurobiological processes, such as higher number of newborn cells in the dentate gyrus, have been involved in the expression of the beneficial effects of EC on long-term object recognition memory [13]. Here, we tried to understand the mechanisms underlying the functional effects of EC on memory by examining the neuronal networks elicited by EC during the memory task. Because EC mice are repetitively exposed to different objects in their home-cage, we hypothesized that the brain region's activity related to the first object exploration (*i.e*. during the presentation session) could be modified in EC mice compared to SC ones. Potential modulation of neuronal networks during the presentation session could underlie a better ability to acquire information about objects in EC mice. Similarly, modification of neuronal networks involved during the test session could also contribute to the promnesiant effects of EC. Herein, we examined the neuronal activation related to both the presentation and the test sessions after 24 h-ISI.

### EC induced a reorganization of the neuronal networks elicited by the presentation and the test sessions of an episodic-like memory task

Immediate-early gene expression analysis is classically used as a neuronal activation marker [45], [46]. More specifically, c-Fos protein expression has been associated with novelty exposure, as well as learning and memory processes such as those occurring during object exploration [21], [28], [47], [48].

Analysis of the neuronal activation profile related to the presentation session revealed a significant activation of the hippocampus and perirhinal cortex in SC mice. In line with the studies of Soule and colleagues [31], our results indicate an activation of hippocampal structures during acquisition of information related to the objects. A similar profile of c-Fos expression was reported after object exposure in a novel environment [49]. By contrast, EC mice did not present hippocampal activation during object exposure at the presentation session. We hypothesize that this might be related to a habituation to novelty and/or repetitive object rearrangement exposure in environmental enrichment. In agreement with this interpretation, rats which experience novel objects for the first time present a relative increased hippocampal activation compared to those repeating the experience and becoming therefore familiar to object exploration [50]. New experiences using EC could lead to a faster hippocampal processing of information acquisition related to the objects. In line with this hypothesis, some authors suggest that exposure to EC may promote the erasure of memory trace in the hippocampus and therefore facilitate new information acquisition [51]. Given the importance of this structure in the novel object recognition task, we examined in a selected fashion the connectivity of the hippocampus with other structures during the task on both the SC and EC mice. We found that hippocampal activity during the presentation session was significantly correlated with more structures in EC than in SC mice (three over six examined and one over six, respectively), especially with the prefrontal regions (prelimbic, infralimbic and anterior cingulate cortex). Whereas the integrity of prefrontal cortex seems not to be required for novel object discrimination [18], [52], [53], higher activation of prefrontal cortex has been reported following object recognition tasks [22]. Here, one could suppose that prefrontal regions take part in the neuronal networks leading to an improved acquisition of information related to the objects in EC mice. A faster cortical transfer of the information related to the objects from the hippocampus may occur in EC mice during the presentation session, in association with an absence of neuronal activation in this last region.

The perirhinal cortex is particularly involved in novel object recognition processes as suggested by lesion studies [18], [54]. Here, we report the activation of this structure during the presentation session in SC mice. This result is in accordance with its reported role in information acquisition related to the objects [47]. Interestingly, we did not find such activation in EC mice. As previously hypothesized in the hippocampus, we could suppose that repetitive exposure to EC housing modifies the neuronal networks related to object exploration in a novel context.

As already reported for aversive memory tasks [6], [55], we show here a significantly reduced activation of the baso-lateral amygdala in EC mice during presentation session, that does not involve explicit emotional arousal. As baso-lateral amygdala is involved in emotional information processing [56], [57], one could suppose that EC enhances the animal's ability to regulate emotional behavior during acquisition of information about an object, a phenomenon that could contribute to memory improvement. In accordance with this hypothesis, it has been recently reported that the baso-lateral amygdala is involved in the formation of long-term memory in a low-arousal novel object recognition task [58].

In addition to its effects during the presentation session, EC modulates the neuronal activation profile related to the test session. We indeed report a significant activation of the infralimbic cortex (belonging to the prefrontal cortex) and of the hippocampus in EC mice during the novel object discrimination, whereas no such activation is found in SC mice. Interestingly, a similar activation profile in the prefrontal cortex and hippocampus was recently reported during novel object discrimination in mice [22]. As prefrontal cortex activation is particularly sensitive to novelty exposure [49], the activation of the prefrontal cortex found only in the EC animals is not surprising, because only the animals of this housing condition discriminated the novel object effectively. The memory trace prolongation in our EC mice seems also to be associated with higher activation of the hippocampus. In the literature, the involvement of the hippocampus seems to be more sensitive to the spatial arrangement of the object, whereas perirhinal cortex activation is more associated with the nature (familiar/novel) of the object [48], [54], [59]. In opposition with Winters and colleagues who attempted to minimize the spatial and contextual factors of the task by using a Y-shaped apparatus with high walls [37], the extra-maze spatial or contextual cues can help our mice identify the arm containing the novel object. Based on the potential influence of these spatial components, the memory task we used could particularly solicit the hippocampus, leading thus to the hippocampal activation found in EC mice. Again, the activity of half of the brain structures analyzed was correlated with the activity of the hippocampus in EC mice, suggesting a major role of this brain structure in the novel object discrimination task in these animals. Finally, we show for the first time a functional neuronal activation of the hippocampus that is associated with novel object discrimination improvement by EC. This finding might be put together with the known hippocampal morphological changes induced by environmental enrichment, such as neurogenesis or synaptogenesis stimulation (see for review [3]). Whether similar hippocampal activation occurs in SC mice during successful novel object discrimination (*i.e.* with 2 h-ISI) remains to be further determined. These investigations could provide evidence for alternative neuronal networks solicited during memory recall in EC mice.

## Conclusions

To conclude, our results confirm previous data supporting the beneficial effects of EC on episodic-like memory persistence in a particular object recognition paradigm in mice. In addition, we demonstrate for the first time that EC exposure modifies the neuronal networks associated with this task of novel object discrimination. In particular, hippocampal activity modulation during both presentation and test sessions may play a pivotal role in the neural circuits engaged in memory improvement by environmental enrichment. Molecular mechanisms underlying these modulations, with a particular interest on the hippocampus, need therefore to be examined. These investigations highlight the relevance of EC to elucidate the brain networks underlying memory plasticity and could help to identify new therapeutic targets in order to alleviate memory deficits such as those consecutive to ageing or neurodegenerative disorders.
